# Fisetin attenuates glutamate-induced excitotoxicity and modulates neural progenitor cell dynamics in vitro

**DOI:** 10.1007/s00210-026-05702-1

**Published:** 2026-07-20

**Authors:** Sergio Martínez-Ortega, Bruno I. Benincasa, Felipe G. Nishimura, Ana L. Fachin, Renata Dellalibera-Joviliano, Pedro Nunez-Abades, Carmen Castro, Rene O. Beleboni

**Affiliations:** 1 Area de Fisiología, Facultad de Medicina, Universidad de Cádiz, Cadiz, Spain; 2 Instituto de Investigación E Innovación Biomedica de Cádiz, Cadiz, Spain; 3 Departmento de Fisiología, Facultad de Farmacia, Universidad de Sevilla, Seville, Spain; 4 Department of Biotechnology, Ribeirão Preto University, Ribeirão Preto, Brazil; 5 Medicine School, University of Ribeirão Preto, Ribeirão Preto, Brazil; 6 Universidade Do Estado de Minas Gerais, Belo Horizonte, Brazil; 7 Carlos Chagas Institute, FIOCRUZ, Curitiba, Brazil

**Keywords:** Fisetin, Neurodegeneration, Excitotoxicity, ROS, Neuroinflammation, Flavonoids, Antioxidant, Anti-inflammatory effects

## Abstract

Neurodegenerative diseases (NDs) are characterized by progressive neuronal loss associated with excitotoxicity, oxidative stress, neuroinflammation, and impaired neuroplasticity. Fisetin, a naturally occurring flavonoid, has emerged as a potential neuroprotective compound due to its antioxidant and anti-inflammatory properties. In this study, we investigated the effects of fisetin in cellular models of glutamate-induced excitotoxicity and neural progenitor differentiation. Differentiated PC-12 cells were exposed to 13 mM glutamate and treated with fisetin. Cell viability was assessed using the MTT assay, reactive oxygen species (ROS) production by dihydroethidium assay, and TNF-α levels by ELISA. In parallel, subventricular zone (SVZ)-derived neurosphere cultures were used to evaluate proliferation and differentiation. Fisetin significantly attenuated glutamate-induced cytotoxicity, reduced ROS production, and decreased TNF-α levels. In neurosphere cultures, fisetin reduced neurosphere size without altering neurosphere number, suggesting modulation of progenitor cell dynamics without affecting neural stem cell self-renewal. Moreover, fisetin increased β-III-tubulin-positive neuroblasts without affecting GFAP-positive glial cells. These findings support the pharmacological potential of fisetin in modulating key pathological features of NDs and encourage further in vivo validation and molecular pathway analysis.

## Introduction

Neurodegenerative diseases(NDs) are characterized by progressive neuronal dysfunction and loss, frequently associated with glutamate-mediated excitotoxicity, oxidative stress, and neuroinflammation. Excessive glutamatergic stimulation promotes reactive oxygen species (ROS) production and inflammatory signaling, contributing to neuronal damage and impairment of neuroplasticity-related processes (Lau and Tymianski 2010; Iovino et al. 2020; Agnello and Ciaccio 2022). Aging is a primary risk for the onset of these disorders, contributing to the development of conditions such as Alzheimer’s and Parkinson’s diseases (Marcos-Rabal et al. 2021; Temple 2023).

Neurogenesis, the process by which new neurons are generated from neural stem cells (NSCs), has been proposed as a potential compensatory mechanism for neuronal loss in neurodegenerative conditions. Under physiological conditions, adult neurogenesis occurs predominantly in specific brain regions, such as the dentate gyrus of the hippocampus (DG) and the subventricular zone (SVZ) (Culig et al. 2022; Wu and Zhang 2023). However, neuroinflammatory mediators, particularly tumor necrosis factor-alpha (TNF-α), can impair the migration and survival of newly formed neurons by activating inflammatory pathways at lesion sites, further exacerbating neuronal dysfunction and damage (Brymer et al. 2018; Uzzan and Azab 2021; Mehterov et al. 2022).

The development of therapeutic strategies capable of reducing oxidative stress and neuroinflammation while promoting neuronal resilience has gained increasing interest. Flavonoids are naturally occurring polyphenolic compounds widely recognized for their antioxidant and anti-inflammatory properties (Ambriz-Pérez et al. 2016). Among them, fisetin has demonstrated neuroprotective potential in different experimental models, including the modulation of oxidative stress and neuronal survival pathways pathways (Elsallabi et al. 2022; Khatoon et al. 2023).

In this study, we investigated the effects of fisetin in vitro using a model of glutamate-induced excitotoxicity and neural progenitor cell differentiation. We evaluated its effects on cell viability, ROS production, TNF-α expression, and differentiation of SVZ-derived neural progenitor cells.

The present study was designed to investigate two complementary pharmacological properties of fisetin. First, using a well-established glutamate-induced injury model in PC-12 cells, we evaluated its effects on cell viability, ROS production, and TNF-α expression. Second, using primary neural stem/progenitor cells isolated from the adult SVZ, we examined whether fisetin directly modulated neural progenitor physiology by regulating proliferation and neuronal differentiation.

## Materials and methods

### Animal subjects

Seven-day postnatal (P7) CD1 mice were used for the isolation of cells from the SVZ. Animals were housed under controlled conditions of temperature (21–23 °C) and light (LD 12:12) with free access to food (AO4 standard maintenance diet; SAFE, Épinay-sur-Orge, France), and water. Care and handling of animals were performed according to the Guidelines of the European Union Council (2010/63/EU) and following the Spanish regulations (65/2012 and RD53/2013) for the use of laboratory animals.

### SVZ cell isolation and culture

For each experiment, cells were obtained from the SVZ of 6 CD1 male and female mice at postnatal day 7. Animals were maintained under controlled housing conditions (21–23 °C; 12:12-h light/dark cycle) with food (AO4 maintenance diet, SAFE, Épinay-sur-Orge, France) and water available ad libitum. All procedures involving animals complied with the European Directive 2010/63/EU and Spanish legislation (65/2012) and RD53/2013) governing experimental animal care and use.

Isolation of cells from the SVZ followed the method described by Rabaneda et al. (2008, 2016). Cells were grown as neurospheres in a defined culture medium consisting of Dulbecco’s Modified Eagle’s Medium/F12 (1:1, v/v) supplemented with 1 mg/L gentamicin (GIBCO) and B27 (Invitrogen, Carlsbad, CA, USA). For expansion, cultures were supplemented with epidermal growth factor (EGF, 20 ng/mL) and basic fibroblast growth factor (bFGF, 10 ng/mL), both obtained from PeproTech (Frankfurt, Germany). Cells were grown as neurosphere aggregates.

### Neurosphere proliferation assay

Cells were isolated from the subventricular zone of P7 (male and female) mice and cultured as neurospheres as explained below. Neurospheres were mechanically dissociated into single cells and seeded in anti-adherent 96-well plates at 2 × 10^4^cells/mL in defined medium containing 10 ng/mL bFGF. Cultures were then exposed to the test compounds. Neurosphere cultures were maintained in defined medium (DM) composed of DMEM/F-12 (1:1) plus the medium supplement B27 (Invitrogen; Carlsbad CA USA). bFGF (10 ng/mL) was added unless otherwise indicated. Neurosphere cells were mechanically disaggregated in DM. Following indications of Curtis and Abernathy (2015b), and based on previous reports (Dominguez-Garcia et al. 2021), results presented here are the average of at least 9 independent experiments.

### Neurosphere formation assays

The number of newly formed neurospheres/well was counted under phase microscopy 72 h after seeding. To measure neurosphere size, images of 10 fields/well were obtained, and the area of the newly formed neurospheres (at least 50 neurospheres/well were measured) was estimated using FIJI software.

### Neurosphere differentiation assay

Neurospheres were collected, centrifuged, disaggregated, seeded onto poly-L-ornithine coated plates, and resuspended in growth factor–free defined medium consistent composed of DMEM/F-12 (1:1) plus the medium supplement B27 (Invitrogen; Carlsbad, CA, USA) in the absence of growth factors. Cells were seeded in the presence of fisetin and maintained for 72 h before fixation for immunocytochemical analysis, as previously reported (Dominguez-Garcia et al. 2020). Following indications of Curtis and Abernathy (2015b), and based on previous reports (Dominguez-Garcia et al. 2020), data were derived from nine independent experiments.

### Immunocytochemistry

Immunolabeling of neuronal and glial markers was conducted as described earlier (Flores-Giubi et al. 2019; Ezzanad et al. 2021b; Pardillo-Díaz et al. 2025). Primary antibodies used were mouse monoclonal anti-β-III-tubulin (1:1000, Cell Signaling Technology, Boston, MA, USA) and rabbit polyclonal anti-GFAP (1:3000, Dako, Hamburg, Germany). Secondary antibodies consisted of donkey anti-rabbit IgG conjugated to Alexa Fluor® 488 (1:1000) and donkey anti-mouse IgG conjugated to Alexa Fluor® 594 (1:1000) (Invitrogen, Carlsbad, CA, USA). Samples were anonymized prior to staining, and blinded quantification of labeled cells was carried out across nine independent experiments.

### Cell culture

The PC-12 cell line (ATCC No. CRL-1721) was cultured in Dulbecco’s Modified Eagle Medium (DMEM) supplemented with 10% fetal bovine serum (FBS). Additionally, an antibiotic cocktail containing 100 I.U./mL penicillin, 100 µg/mL streptomycin, and 50 µg/mL kanamycin was included. Cells were maintained in a humidified incubator at 37 °C with 5% CO₂ until reaching ~ 5 × 10⁶ cells, at which point subculturing was performed to maintain optimal growth conditions. Additionally, prior to cell viability and ROS assays, PC-12 cells were treated with 100 ng/mL of nerve growth factor (NGF) derived from *Vipera lebetina*venom (Sigma-Aldrich, N8133) and incubated for 6 days (144 h) to induce neuronal-like differentiation (Sierra-Fonseca et al. 2014; Hu et al. 2018).

### Cell viability assays

Cell viability was assessed using the MTT (3-[4,5-dimethylthiazol-2-yl]−2,5-diphenyl tetrazolium bromide) assay. PC-12 cells were seeded in 96-well plates at a density of 5 × 10^4^cells/well, with a final medium volume of 200 µL per well. After cell differentiation with NGF, cells were treated with 13 mM glutamate or 13 mM glutamate + different concentrations of fisetin (0.50, 1.00, 1.95, 3.9, 7.8, 15.6 µM). Concentrations were chosen based on previous reports and effective pilot assays (Chang et al. 2023; Ji et al. 2024). For the negative control, the culture medium contained 0.2% DMSO.

### Reactive oxygen species

The dihydroethidium (DHE) assay was employed for ROS detection (Zhu et al. 2024). Cells were seeded at a density of 1 × 10⁶ cells per well in 6-well plates. After cell differentiation, the wells were assigned to the following groups: positive control (13 mM glutamate), negative control (culture medium with 0.2% DMSO), and treatment with fisetin at 7.8 µM.

### TNF-α dosage

After treatment, culture supernatants from each experimental group (positive control 13 mM glutamate, negative control 0.2% DMSO, and fisetin 7.8 µM) were collected for TNF-α quantification. TNF-α levels were measured using the Rat TNF-α ELISA kit (Sigma-Aldrich, RAB0479), containing modifications (Mattos et al. 2020; Dellalibera-Joviliano et al. 2024).

### Statistical analysis

All experimental data were expressed as mean ± standard error of the mean (SEM). Comparisons between multiple treatment groups were performed using one-way analysis of variance (ANOVA), followed by post hoc test for pairwise comparisons. A *p*-value of less than 0.05 was considered statistically significant.

For neurosphere proliferation and differentiation assays, the data and statistical analysis comply with the recommendations on experimental design and analysis in pharmacology (Curtis and Abernethy 2015a). Statistical analyses were performed using one-way ANOVA followed by a post hoc test unless otherwise indicated. Differences were considered significant at values of*p* < 0.05. In general, the sample size used in statistical analysis was *n*= 6–10 for in vivo experiments. Sample sizes were chosen based on previous works related to this one  (Murillo-Carretero et al. 2017; Dominguez-Garcia et al. 2020, 2021); Ezzanad et al. 2021a; Gómez-Oliva et al. 2023a, b).

## Results

### Fisetin shifts SVZ neurosphere dynamics toward differentiation-like profiles

**Fig. 1:**
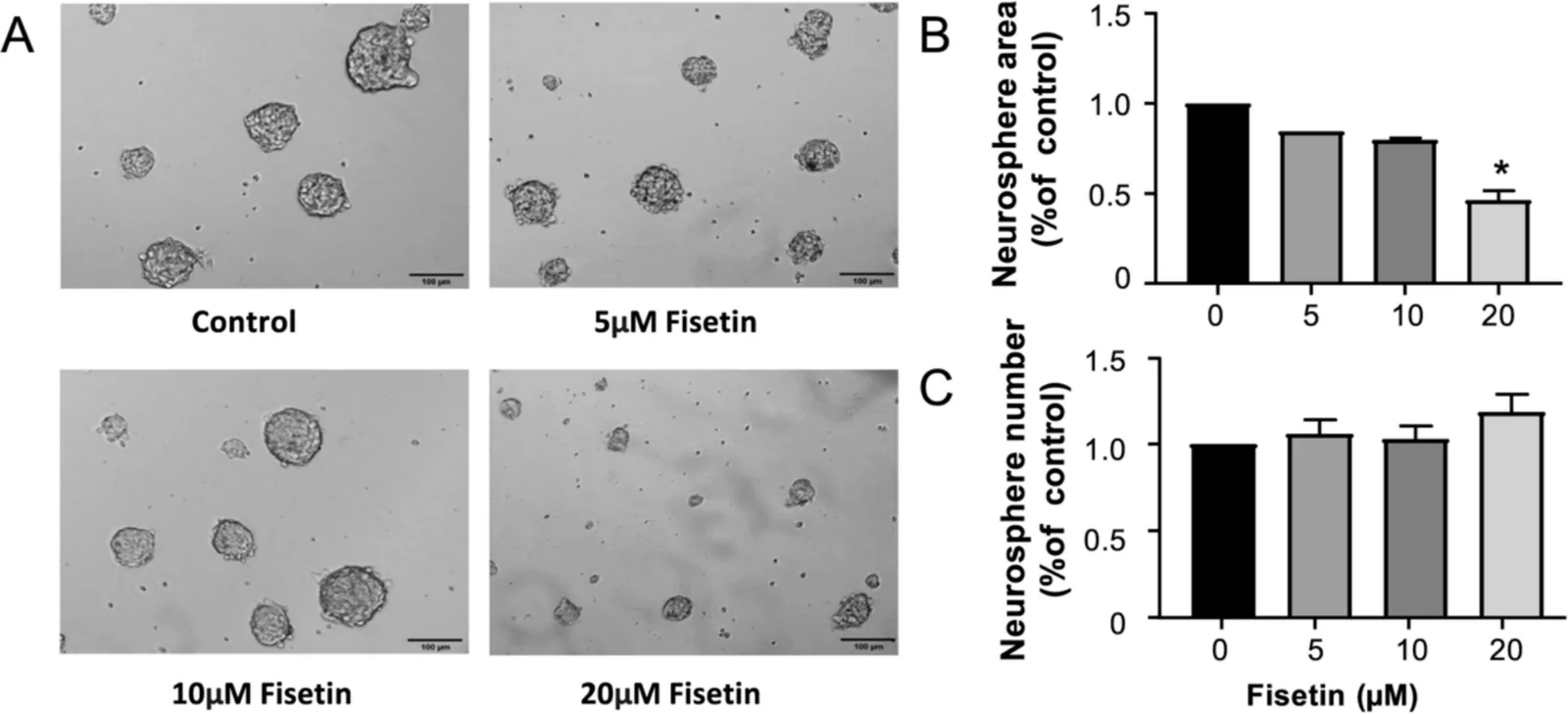
Fisetin inhibits proliferation of SVZ-derived cells.** A** Representative phase microscopy image of the neurospheres formed from SVZ-isolated cells grown for 72 h in the presence of EGF + bFGF (control) or in the combined presence of EGF + bFGF and increasing concentrations of fisetin (5, 10, and 20 µM). **B** Dose–response: neurosphere size in the presence of increasing concentrations of fisetin. **C** Dose response: neurosphere number in the presence of increasing concentrations of fisetin. Data are the mean ± SEM of nine independent values. Differences were detected by one-way ANOVA followed by the Tukey b test. (*p < 0.05 vs the rest of groups) Scale bar = 100 µm

To determine whether fisetin influences neural stem cell physiology, we evaluated both their proliferative and differentiative capacity (Fig. 1). The analysis showed no significant difference in neurosphere number compared with controls (Fig. 1A, C), indicating that fisetin does not affect the self-renewal capacity of neurosphere-forming cells (neural stem cells or undifferentiated progenitors). In contrast, fisetin significantly reduced neurosphere size, indicating reduced expansion of progenitor-derived cells (Fig. 1A, B).

**Fig. 2:**
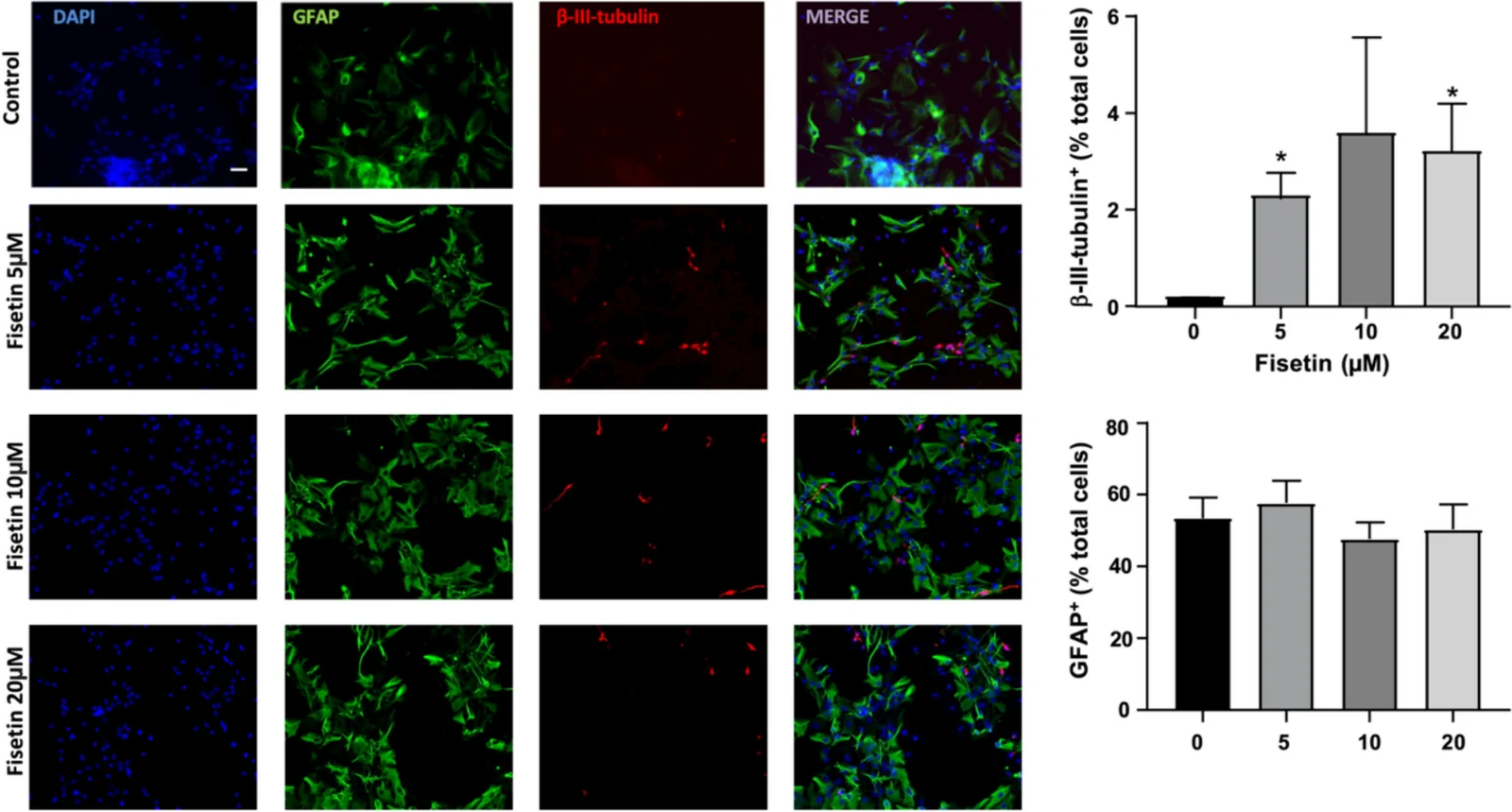
Fisetin promotes differentiation of SVZ-isolated progenitor cells to a neuronal phenotype. **A** Representative fluorescence images of SVZ-derived cultured NPC that had been treated with either diluent (control) or increasing concentrations of fisetin (5, 10, and 20 µM). Cells were grown without growth factors, allowed to differentiate for 72 h after treatment, and then fixed. β-III-tubulin marker was used for neuronal immunodetection (red), and glial cells were identified by the immunodetection of GFAP (green). Total nuclei were counterstained with DAPI (blue). **B** Graph represents the percentage of total cells (detected by DAPI nuclear staining) that were positive for β-III-tubulin. **C** Graph represents the percentage of total cells (detected by DAPI nuclear staining) that were positive for GFAP. Data are the mean ± SEM of nine independent values (*n* = 9). Differences were detected by one-way ANOVA followed by the Tukey b test (*p < 0.05 vs control). Scale bar: 100 µM

In the absence of fisetin (control), cells differentiated mainly into astrocytes (Fig. 2). Quantification of β-III-Tubulin expression indicated that fisetin significantly increased the number of cells that expressed this marker, suggesting a role in facilitating differentiation into a neuronal phenotype. In contrast, analysis of GFAP expression revealed no statistically significant differences between fisetin-treated groups and the negative control, suggesting that fisetin promotes neuronal differentiation without inducing glial cell differentiation, supporting its potential role in neurogenic processes.

### Fisetin attenuates glutamate-induced excitotoxicity

**Fig. 3:**
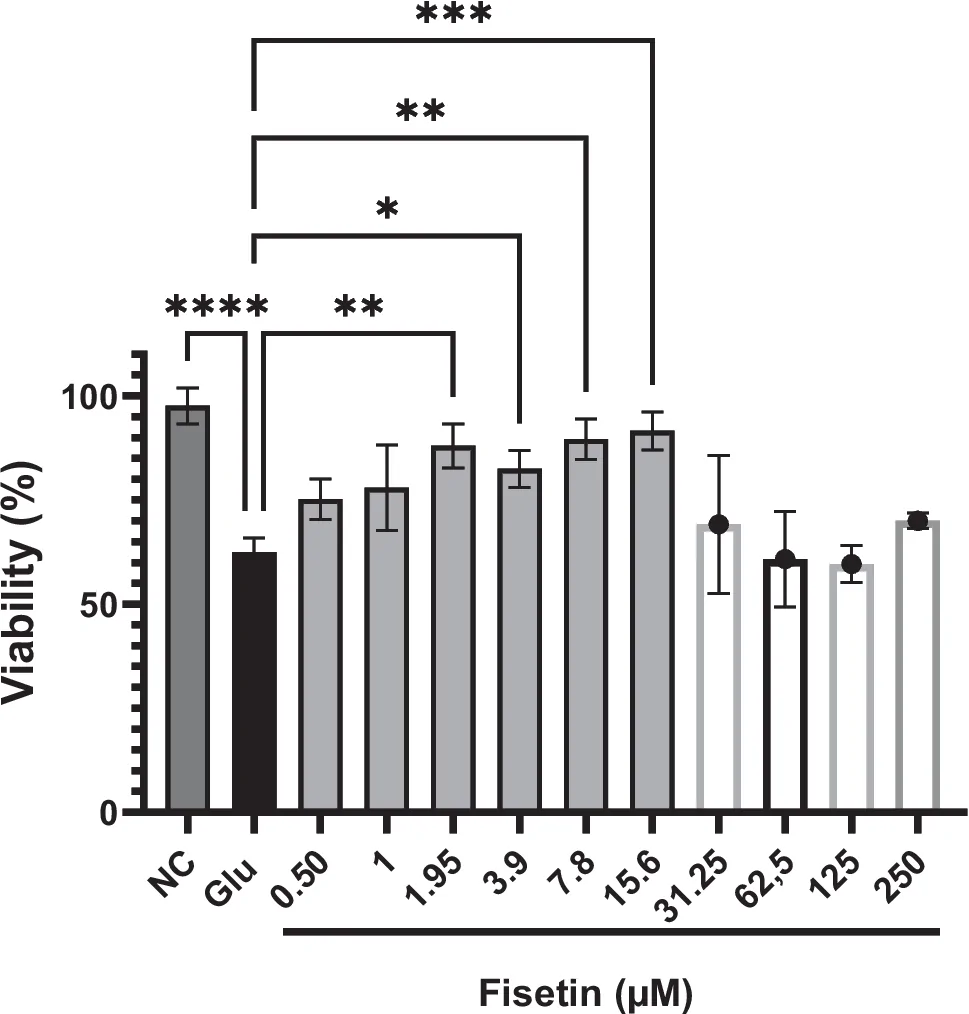
Cell viability in response to glutamate-induced excitotoxicity and fisetin cytoprotection. Differentiated PC-12 cells were exposed to glutamate followed by fisetin treatment to assess its potential cytoprotective effect. The glutamate-only condition (Glu) and a negative control (NC) were included as controls. ***p < 0.001, ****p < 0.0001

Glutamate-induced excitotoxicity in differentiated PC-12 cells has been previously reported within a concentration range of 0.01–30 mM (Wang et al. 2010; Lin et al. 2014). Based on this last evidence and on pilot assays (not published), 13 mM glutamate treatment for 24 h was identified as optimal concentration for inducing significant cellular damage while maintaining a measurable population of viable cells for further analysis. Fisetin significantly decreased glutamate-induced cytotoxicity at 1.95 µM, 7.8 µM, and 15.6 µM, preserving cell viability (Fig.3). Since the range of significant differences was between 1.95 µM and 15.6 µM, the intermediate dose of 7.8 µM was selected as the treatment concentration for the subsequent experiments for %ROS and TNF-α. Higher concentrations (250, 125, 62.5, and 31.25 µM) were also tested; however, because these doses were inherently cytotoxic, they were deemed unsuitable for further evaluation.

**Fig. 4:**
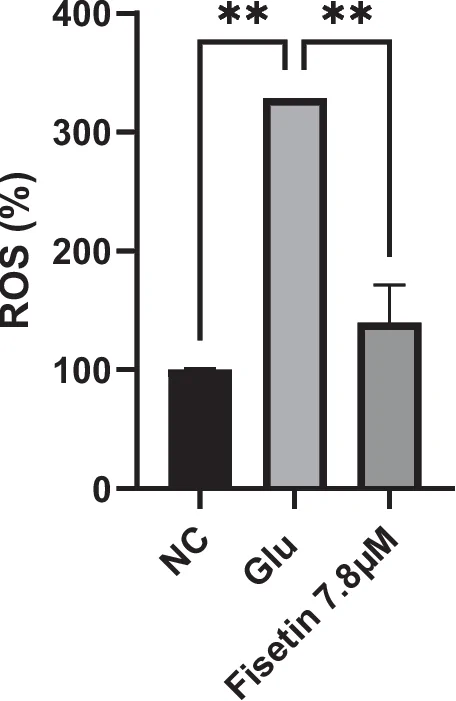
Measurement of reactive oxygen species (ROS) levels. Cells were exposed to 13 mM glutamate to induce ROS production, followed by treatment with fisetin to evaluate its antioxidant effects. The experimental groups included negative control (NC), a glutamate-only condition (Glu), and fisetin-treated cells. Data are expressed relative to the NC group (set as 100%). **p < 0.005

The negative control was set as 100% to provide a baseline for comparison in measurement of ROS production by DHE assay (Fig. 4). Treatment with 13 mM glutamate resulted in a marked increase in ROS production. Treatment with fisetin significantly decreased ROS production relative to the glutamate-only group, and no statistically significant difference was observed between the fisetin-treated and control groups, suggesting that fisetin may help preserve ROS levels within a physiological range and potentially confer protection against glutamate-induced oxidative stress.

**Fig. 5:**
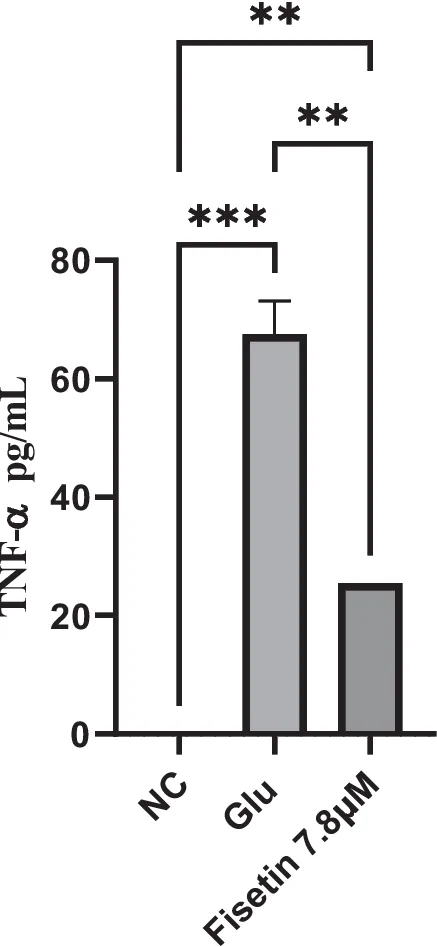
Quantification of TNF-α levels by ELISA following glutamate-induced excitotoxicity and fisetin treatment. TNF-α concentrations were measured in the culture supernatants of differentiated PC-12 cells under three conditions: negative control (NC), glutamate treatment (Glu), and fisetin (7.8 µM). **p < 0.01, ***p < 0.001

Similarly, as shown in Fig. 5, Glu treatment significantly increased TNF-α expression compared to NC, confirming its pro-inflammatory effect. Treatment with fisetin significantly reduced TNF-α expression compared to Glutamate alone, indicating that fisetin effectively counteracts the Glu-induced upregulation of this cytokine. Furthermore, TNF-α levels in the fisetin-treated group remained significantly elevated compared to NC, suggesting a partial, but not complete, restoration to basal inflammatory levels.

## Discussion

Our findings demonstrated that fisetin exerts a significant cytoprotective effect in neuron-like cells subjected to glutamate-induced excitotoxicity. The MTT viability assay showed that fisetin effectively preserved cell viability. This is consistent with previous work showing that fisetin prevents corticosterone-induced cytotoxicity in PC-12 cells, attributed to its antioxidant action through reduction of ROS (Chang et al. 2023).

Glutamate-induced oxidative stress led to a robust increase in ROS production, which was markedly mitigated by fisetin treatment. Like other flavonoids, fisetin exhibits notable ROS-scavenging capacity. Its antioxidant effect has been previously linked to the stabilization and activation of the Nrf2–ARE signaling pathway and downstream cytoprotective genes such as HO-1 (Maher 2006; Chen et al. 2015; Yen et al. 2017; Zhang et al. 2019). These findings further corroborate fisetin’s role in restoring redox homeostasis under excitotoxic conditions. Importantly, the glutamate-treated PC-12 model was specifically used to evaluate the neuroprotective, antioxidant, and anti-inflammatory properties of fisetin under conditions of oxidative stress, whereas the primary SVZ-derived cultures were used to investigate whether fisetin directly modulated neural stem/progenitor cell behavior under basal conditions.

Notably, fisetin also presented an anti-inflammatory effect, evidenced by the significant reduction of TNF-α expression in glutamate-exposed cultures. TNF-α is a pro-inflammatory cytokine widely implicated in neurodegenerative and neuroinflammation process (Adamu et al. 2024). Inflammation is commonly associated with increased glutamate levels (Haroon et al. 2016b), but here glutamate robustly increased TNF-α levels, which is consistent with the close relationship between these processes  (Haroon et al. 2016a). Remarkably, fisetin treatment significantly downregulated TNF-α expression, suggesting it effectively blunts inflammatory signaling triggered by excitotoxicity.

Interestingly, fisetin reduced neurosphere size without affecting neurosphere number. In the neurosphere assay, the number of neurospheres is commonly interpreted as a readout of the presence and self-renewal capacity of neural stem cells (NSCs), and it is generally affected by oxidizing agents like nitric oxide  (Torroglosa et al. 2007). Since in our experiments the number of neurospheres remained unchanged, these results suggest that the stem cell pool was preserved and that neither NSC survival nor their ability to initiate sphere formation was compromised. In contrast, the reduction in neurosphere size indicates an effect on the proliferative expansion of progenitor cells within each sphere. Taken together, these data point to an impairment in progenitor cell expansion without a direct effect on the maintenance of the stem cell population. Since the primary SVZ-derived cultures were not subjected to an oxidative insult, viability and ROS production were not considered primary endpoints in this experimental setting. Instead, these cultures were used to determine whether fisetin directly influenced neural progenitor proliferation and lineage commitment independently of oxidative stress.

Having demonstrated the neuroprotective properties of fisetin in the glutamate-induced injury model, we next examined whether the compound also directly influenced adult neural stem/progenitor cell differentiation. As expected, a positive effect on SVZ cell differentiation was observed in the presence of fisetin. The compound significantly increased neuronal differentiation, as evidenced by the higher percentage of βIII-tubulin-positive cells observed after fisetin treatment, without affecting astrocyte differentiation. It should be noted that the present differentiation protocol was designed to evaluate early neuronal fate commitment rather than neuronal maturation. Accordingly, βIII-tubulin was used as an early neuronal marker, whereas assessment of mature neuronal phenotypes (e.g., MAP2 or NeuN markers) would require longer differentiation periods and falls beyond the scope of the present study. The ability of fisetin to promote neuronal differentiation raises the possibility that it may also influence subsequent stages of neurogenesis, including neuroblast migration, as previously reported for other compounds studied by our group.

The experimental platform used in the present study was specifically designed to interrogate intrinsic neural stem/progenitor cell physiology, focusing on proliferation and differentiation endpoints, and does not reproduce the three-dimensional architecture, guidance cues, or chemotactic gradients required for a meaningful assessment of migration. Future studies using time-lapse single-cell tracking, SVZ explant cultures, or organotypic slice models that preserve directional cues will be necessary to determine whether fisetin directly modulates neuroblast migration in physiologically relevant contexts.

The reduction of TNF-α and ROS by fisetin may help reduce neuroinflammation, as well as create a more favorable environment for neurogenesis. The impact that fisetin might have in neurogenesis is supported by the increase in β-III-tubulin-positive neuroblasts found in our differentiation studies in vitro. Notably, this effect on neuronal differentiation occurred without an associated increase in GFAP-positive glial cells, indicating that fisetin selectively drive neuronal over glial lineage commitment, a desirable outcome in regenerative strategies aimed at neuronal replacement.

Although the present study provides relevant insights into the neuroprotective and neurogenic potential of fisetin, some limitations inherent to in vitro approaches should be acknowledged. First, PC-12 cells, despite being widely used as a neuronal-like model, are derived from a rat pheochromocytoma and do not fully reproduce the molecular and metabolic complexity of primary neurons or mature neuronal networks. Therefore, the observed cytoprotective effects of fisetin should be interpreted as indicative rather than fully representative of neuronal behavior in vivo.

Consequently, although our data support a neuroprotective, antioxidant, and anti-inflammatory role for fisetin, further in vivo studies will be essential to validate these effects under physiological and pathological conditions, as well as to determine their functional relevance in animal models of neurodegenerative diseases.

Nevertheless, these results suggest that fisetin is a promising therapeutic candidate that reduces key pathophysiological features of neurodegeneration, such as excitotoxicity, oxidative stress, and impaired neurogenesis, while preserving glial homeostasis. Future studies should explore its in vivo efficacy. Additionally, transcriptomic or proteomic profiling could elucidate the molecular networks modulated by fisetin, revealing potential synergistic targets for combinatorial therapies and mechanisms behind such effects.

## Data Availability

No datasets were generated or analysed during the current study.
